# Regarding: ‘Perceptions of research experience during internal medicine training: insights from a national survey’

**DOI:** 10.1080/07853890.2025.2561798

**Published:** 2025-09-17

**Authors:** Tuana Haznedar, Doga Aksoy

**Affiliations:** ^a^ Oxford University Hospitals NHS Foundation Trust, Oxford, Oxfordshire, UK; ^b^ Wye Valley NHS Trust, Hereford, Hereforshire, UK

Dear Editor

We read the article by US colleagues on the recent study exploring internal medicine (IM) resident’s perceptions of research [1] with interest. The study highlights concerns that are uncomfortably familiar in the UK. Dissatisfaction with research opportunities, the negative impact of publication pressure on well-being, and doubts about the quality of academic outputs has become a global problem.

In the United Kingdom, Internal Medicine Training (IMT) recruitment has recently revised its application scoring matrix to promote fairness and achievability [2]. While well intended, narrowing the domains has concentrated competition in the few areas that remain. Publications continue to carry disproportionate weight, creating pressure to ‘produce something’ even in the absence of meaningful time, training, or supervision.

The consequences are strikingly similar on both sides of the Atlantic. Recycled audits, low-impact presentations, and inflated authorship are increasingly common, not as a reflection of trainee’s integrity but of structural incentives that reward output over substance. Research risks becoming an exercise in point scoring rather than a genuine opportunity to contribute to science or improve patient care.

This culture determines something far more important, clinical excellence. Safe, thoughtful, and effective patient care is the core of medicine, yet in recruitment processes it is often assumed rather than actively rewarded. A trainee who consistently delivers outstanding clinical work, demonstrates excellent judgement, and provides high quality care to patients may find this counts for less than a token publication. This imbalance sends the wrong message, that academic output is a prerequisite for career progression, while excellence in clinical practice is insufficient on its own.

Realigning priorities is essential. Research should be supported with mentorship, time, and resources for those genuinely interested, but should not be forced on all trainees. Clinical excellence, teaching, leadership, and service innovation should be explicitly valued and rewarded within selection processes. To neglect this is to risk losing focus on developing doctors who provide the best possible care for patients.

The findings from the US survey act as a warning. Unless expectations are reset, publication pressure will continue to distort postgraduate training. We must ensure that clinical excellence is not overshadowed by the drive to publish.

## Data Availability

No new data were generated or analyzed in support of this correspondence. Data sharing is not applicable to this article.
